# Facile synthesis of high-surface-area nanoporous carbon from biomass resources and its application in supercapacitors†

**DOI:** 10.1039/c7ra12525a

**Published:** 2018-01-09

**Authors:** Yuechao Yao, Qi Zhang, Peng Liu, Liang Yu, Lin Huang, Shao-Zhong Zeng, Lijia Liu, Xierong Zeng, Jizhao Zou

**Affiliations:** Shenzhen Key Laboratory of Special Functional Materials, Shenzhen Engineering Laboratory for Advance Technology of Ceramics, College of Materials Science and Engineering, Shenzhen University Shenzhen 518060 P. R. China zoujizhao@szu.edu.cn; Key Laboratory of Optoelectronic Devices and Systems of Ministry of Education and Guangdong Province, College of Optoelectronic Engineering, Shenzhen University Shenzhen 518060 P. R. China; School of Aerospace, Transport and Manufacturing, Cranfield University Cranfield Bedfordshire MK43 0AL UK

## Abstract

It is critical for nanoporous carbons to have a large surface area, and low cost and be readily available for challenging energy and environmental issues. The pursuit of all three characteristics, particularly large surface area, is a formidable challenge because traditional methods to produce porous carbon materials with a high surface area are complicated and expensive, frequently resulting in pollution (commonly from the activation process). Here we report a facile method to synthesize nanoporous carbon materials with a high surface area of up to 1234 m^2^ g^−1^ and an average pore diameter of 0.88 nm through a simple carbonization procedure with carefully selected carbon precursors (biomass material) and carbonization conditions. It is the high surface area that leads to a high capacitance (up to 213 F g^−1^ at 0.1 A g^−1^) and a stable cycle performance (6.6% loss over 12 000 cycles) as shown in a three-electrode cell. Furthermore, the high capacitance (107 F g^−1^ at 0.1 A g^−1^) can be obtained in a supercapacitor device. This facile approach may open a door for the preparation of high surface area porous carbons for energy storage.

## Abstract 2

High-surface-area nanoporous carbon is obtained by direct pyrolysis of biomass resources without an activation process. An electrochemical test shows high capacitance.

## Graphical abstract

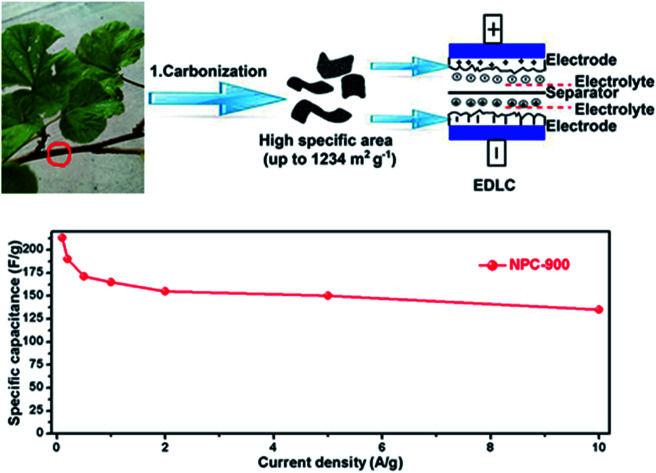

## 1. Introduction

Supercapacitors have been used in energy storage devices and have been a hot research area, due to their long cycle life and high power density.^1–3^ Supercapacitors can be divided into electrical double layer capacitors (EDLC) and Faraday capacitors (pseudocapacitor). EDLCs store the charge electrostatically by reversible desorption–adsorption of ions at the interface between the electrolyte and active materials. Currently, the main commercially available supercapacitors, EDLC, use carbon-based materials as electrode active materials, due to their extraordinary cycle stability, high power density, easy fabrication and non-toxicity.^4^ Carbon materials, such as nanoporous carbon,^5–7^ graphene,^8–10^ carbon nanotubes,^11,12^ carbon nanofibers,^13–15^*etc.* have been widely used in supercapacitors. The higher capacitance of carbon materials is highly demanded. To provide high capacitance, a large specific surface area (SSA) and high porosity are desired because more electrons can be effectively stored in electric double layer capacitors (EDLC).^16–18^ Moreover, the hydrophilicity of carbon materials is another important factor that affects the electrochemical performance because a poor contact between electrode and electrolyte in the aqueous medium would lead to the reduced effective SSA.^19^

In recent years, many strategies have been reported to obtain nanoporous carbon (NPC) with high SSAs, including carbonization of high SSAs metal–organic frameworks (MOFs),^20,21^ physical chemical activation,^10,22–26^ and nanocasting strategy with hard templates^27^ (for example, nanoporous silica). Although NPC of high SSAs was obtained by above these methods, their processes of production were very complicated and costly.

Lately, the NPC materials from renewable biomass resources through pyrolysis method have attracted much attention due to their sustainability and low cost.^28–32^ Duan *et al.* reported carbon nanofibrous microspheres (the SSA up to 1141 m^2^ g^−1^) derived from renewable chitin.^33^ In the organic electrolyte, the energy density of the carbon nanofibrous microspheres reached up to 58.7 W h kg^−1^ in supercapacitors. Moreover, Liu *et al.* reported a porous carbon fibers by using cotton as carbon precursor *via* the pretreatment of fiber in NaOH/urea solution and a subsequent carbonization process.^34^ These porous carbon fibers exhibit a high electric double layer capacitance (221.7 F g^−1^ at 0.3 A g^−1^). A series of high SSA NPC materials have been obtained from renewable biomass resources through pyrolysis. However, almost all reported processes are complicated and need pre-treatment for the precursors or following activation process. Therefore, a facile method of calcining renewable biomass resources to obtain high SSA NPC materials is highly desired.

**Scheme 1:**
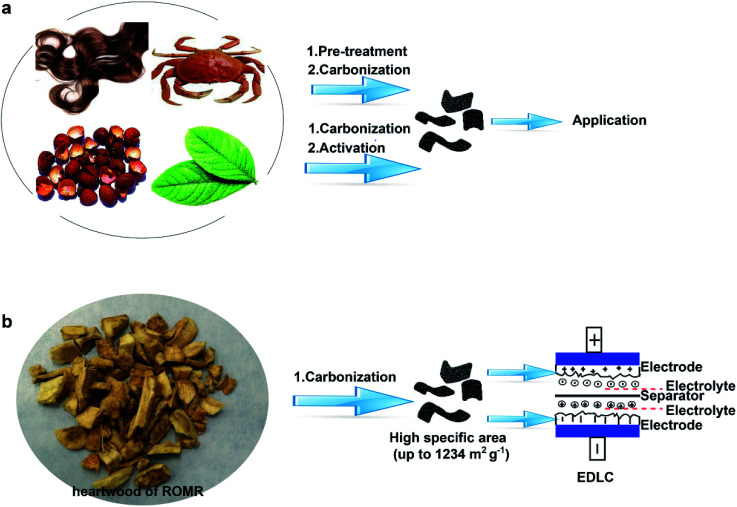
Schematic illustration for the preparation of NPC. (a) Fabrication of conventional NPC by carbonization method. This method usually includes process of pre-treatment or activation. (b) Fabrication of NPC through a facile procedure without other process.

In this study, we reported a facile and cost-effective method to produce NPC materials with high SSA by carbonizing heart wood of Root of Multibract Raspberry (ROMR explanation, S1†). Compared to the other processes for pre-treatment or activation step (Scheme 1a), the high SSA (1234 m^2^ g^−1^) was obtained simply by the direct carbonization of precursor at 900 °C in our process as shown in Scheme 1b. The electrochemical performance of the obtained NPC materials in supercapacitors was systematically studied. The results showed a high capacitance (up to 213 F g^−1^ at 0.1 A g^−1^) and a stable cycle performance (6.6% loss over 12 000 cycles) in a three-electrode cell.

## 2. Experimental section

### 2.1 Synthesis of NPC

The precursor for porous carbon was obtained by extracting the inner component of Root of Multibract Raspberry and was dried in an oven at 100 °C for 24 h. The dried precursor was carbonized in a tubular furnace at 800, 900 and 1000 °C for 2 h at a heating rate of 10 °C min^−1^ under an Ar atmosphere. The obtained carbon materials were treated with 1 mol L^−1^ hydrochloric acid, and washed several times with deionized water and vacuum dried at 120 °C for 24 h. The final products were labeled as NPC-800, NPC-900 and NPC-1000.

### 2.2 Materials characterization

Field emission scanning electron microscopy (FESEM) images were captured by a Hitachi SU-70 FESEM instrument, and transmission electron microscopy (TEM) was conducted *via* a JEOL 2011 TEM facility. X-ray diffraction (XRD) patterns were recorded on a Bruker D8 advance powder X-ray diffractometer using a Cu Kα radiation. Elemental analysis was acquired using a Vario EL cube elemental analyzer. The specific surface areas were determined by the gas sorption technique using a Micromeritics ASAP 2020 based on the Brunauere–Emmette–Teller (BET) method. Laser Raman spectroscopy was performed on a Renishaw inVia Spectrometer. X-ray photoelectron spectroscopy (XPS) was investigated on a ULVAC-PHI 1800 spectrometer. The electrochemical properties were examined on an electrochemistry workstation (CHI660E).

### 2.3 The fabrication of supercapacitor electrode and electrochemical measurements

All electrochemical measurements were carried out in a standard three-electrode electrochemical cell equipped with a Pt counter electrode, an Hg/HgO reference electrode. The NPC electrodes were prepared by mixing 85 wt% active materials, 10 wt% acetyleneblack and 5 wt% polytetrafluoroethylene with ethanol to form slurries that were then spread onto nickel foams with the coating area of 1 cm^2^. These foams were dried at 110 °C under vacuum overnight and then pressed under a pressure of 10 MPa to completely adhere to the electrode materials. The loading mass of active materials on the prepared working electrode is approximately 3 mg cm^−2^.

The electrochemical performances were characterized in a three-electrode system using 6 M KOH as electrolyte, a saturated calomel electrode as reference electrode, and a platinum foil as a counter electrode. CV measurements were performed over the potential range from 0 to −1 V in 6 M KOH, at scan rates of 5, 10, 25, 50 and 100 mV s, respectively. Electrochemical impedance spectroscopy (EIS) measurements were taken over the frequency range from 100 kHz to 0.01 Hz. In the three-electrode configuration, the specific capacitance, *C*_3E_, was calculated from the discharge curve according to eqn (1):

1 *C*_3E_ = *I*Δ*t*/(*m*Δ*V*)

where *C*_3E_ (F g^−1^), *m* (g), *I* (A), Δ*t* (s), Δ*V* (V) are specific capacitance, mass of the active materials in the electrode, discharge current, discharge time, and potential window of gravimetric charge–discharge (GCD) curve, respectively.

The electrochemical performance was also tested in a 2-electrode symmetric supercapacitor configuration. In this configuration, the *C*_2E_ of NPC was calculated based on eqn (2):

2 *C*_2E_ = 4*I*Δ*t*/(*m*Δ*V*)

where *m* is the mass of the active materials in a device.

## 3. Results and discussion

**Fig. 1:**
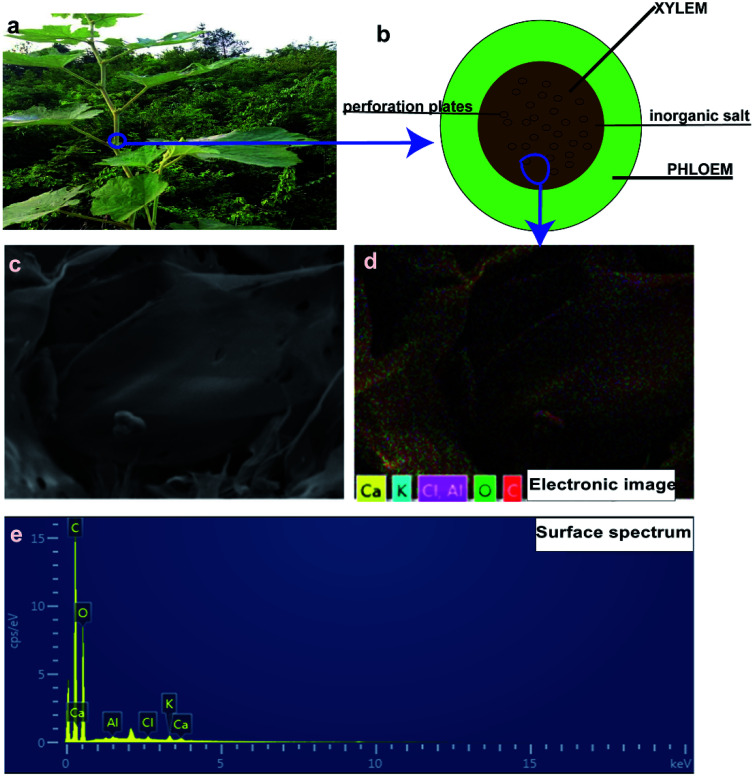
(a) The digital photos of ROMR. (b) The images of internal anatomy for ROMR trunk. (c–e) The EDX results of the calcination precursor.

Fig. 1 illustrates the mechanism for the synthesis of the ROMR (Fig. 1a) derived nanoporous carbon (NPC) by self-activation. The seed plants can be divided into two types, angiosperm and gymnosperms. ROMR belongs to angiosperm, and their trunk is made of xylem and phloem as shown in Fig. 1b. Moreover, there are a large amount of perforation plates in xylem area. This special structure plays a transportation aisle (H_2_O) role in ROMR, and has a lot of pores to store moisture. These pores can be maintained after calcination. The EDX result of xylem was exhibited in Fig. 1c–e, and a lot of inorganic salts (Al, Ca, K) were found. The specific content of Al, Ca, K were investigated by EDX mapping and are 0.16 wt%, 0.87 wt% and 0.49 wt% in precursor, respectively (Table S1†). These meltable salts play an activation role and step up the improvement of SSA in NPC materials.^35–37^ In a word, after calcination the high SSA of NPC materials were attributed to the co-work of special perforation plates and inorganic salts. In view of above two points, the heartwood of ROMR was selected. They are widely distributed in China has fully illustrated that they can get widely application.

**Fig. 2:**
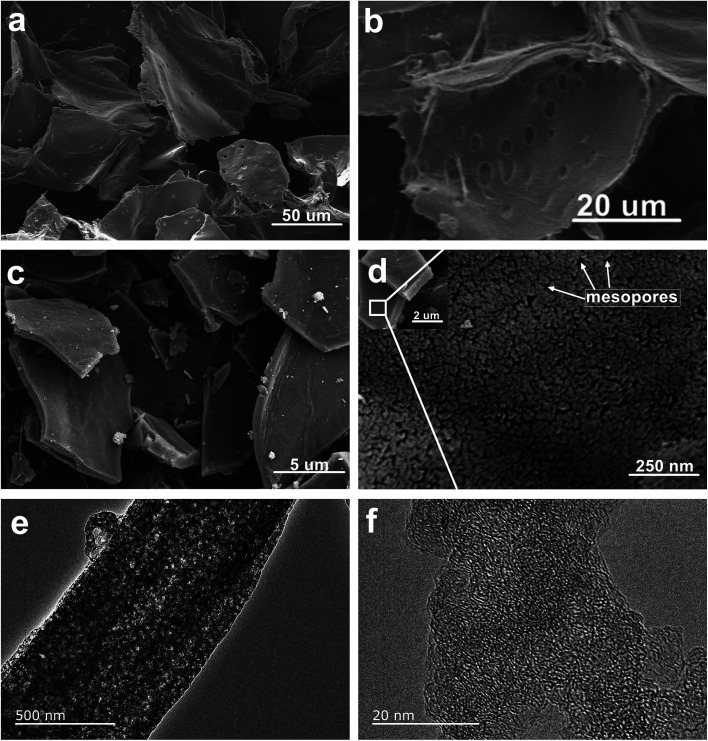
(a and b) SEM images of ROMR trunk inner structure. SEM images (c and d) and TEM (e and f) of NPC-900.

Next, the microstructure of heartwood of ROMR and NPC materials was observed by SEM and TEM. The architecture of precursors before calcination shows a 3D sheet morphology (Fig. 2a). The higher magnification SEM (Fig. 2b) of heartwood of ROMR shows a lot of perforation plates (pores) and corresponding to the Fig. 1b results. After calcination, the SEM images of NPC were showed in Fig. 2c, the 3D sheet structure can be maintained. Moreover the higher magnification SEM is provided in Fig. 2d, the nanoporous carbon was clearly observed, especially mesopores. As shown in Fig. 2e, a lot of bright pores can be clearly observed in NPC. Moreover, the high magnification TEM results of NPC further illustrated these pores (Fig. 2f).

**Fig. 3:**
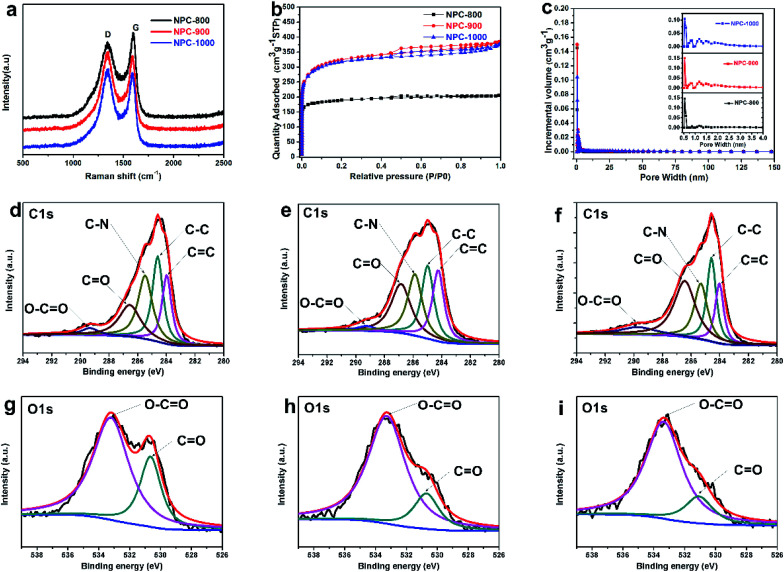
(a) Raman spectrum. (b) Nitrogen adsorption–desorption isotherm. (c) Pore size distribution of NPC-800, NPC-900 and NPC-1000. XPS spectra of (d, e, f) C 1s and (g, h, i) O 1s for NPC-800, NPC-900 and NPC-1000, respectively.

To investigate the influence of different calcination temperature on NPC physical performance, the precursor materials were calcined at 800, 900, and 1000 °C. The XRD patterns of NPC for different calcination temperatures were exhibited in Fig. S2.† The XRD profiles showed two broad peaks located at ∼23° and ∼44° corresponding to the (002) and (100) reflections of carbon materials, respectively. The NPC-800 has a relatively high (002) peak intensity compared to that of others, indicating a higher degree of graphitization in all NPC materials.^38^ In contrast, for the NPC-900 and NPC-1000 samples, the peak at ∼23° became a lower shoulder while a large increase in the low-angle scatter was observed instead, indicating a lot of nanoporous existing.^39^ The results were consistent with the previous TEM. Moreover, the Raman spectrum has further proved the above consequence as shown in Fig. 3a. The graphitic layers are reflected by the G band value, while the D band corresponds to disordered carbon or defective graphitic structures.^29^ The intensity ratio (*I*_D_/*I*_G_) of these two peaks partially depends on the graphitization degree.^40^ The D peak value at ∼1349 cm^−1^ increased drastically with the increases of calcination temperature, indicating more disorder carbon produced. The NPC-800 gets the better graphitization degree with lower *I*_D_/*I*_G_ ratio (0.88). With the increase of pyrolysis temperature, the *I*_D_/*I*_G_ ratio increased to 0.912 (NPC-800) and 1.063 (NPC-1000). The existence of a lot of disorder carbon in NPC-900 and NPC-1000 resulted in high SSA as shown in Table 1. The nitrogen adsorption–desorption measurements were conducted to analyze the detailed pore structure of the NPC materials obtained by calcination at 800, 900 and 1000 °C. The N_2_ absorption analysis of NPC-900 and 1000 shows type-IV curve with a gradual capillary condensation step (hysteresis loop) in the relative pressure range of 0.5–0.8 (Fig. 3b), suggesting the existence of hierarchical mesopores structures.^41^ As shown in Fig. 3c (inset) the pore size distribution shows uniform micropores with average diameters of 0.5 nm and some mesopores with average diameters of 2.5 nm. For NPC-800, 900 and 1000, the SSA were 589, 1234 and 1209 m^2^ g^−1^, respectively. The corresponding pore volume was 0.32, 0.59 and 0.57 cm^3^ g^−1^, respectively. From the XPS investigation of the NPC materials, three elements, carbon, oxygen and nitrogen, were detected (Fig. S3†). The C 1s spectrum of the NPC carbon was fitted using five component peaks with binding energy of∼284.4, ∼284.9, ∼285.7, ∼286.7 and ∼289.2 eV (Fig. 3d–f), attributed to the contribution of C=C, C–C, C–N, C=O and O–C=O, respectively.^33,39^ The high-resolution O 1s spectrum (Fig. 3g–i) can be divided into two peaks located at ∼531.2 and ∼533.5 eV, corresponding to C=O and O–C=O groups. By comparison of Fig. 3g–i, the intensity of C=O group drastically decreased with the calcination temperature, which illustrates a reduction in O-functionalities. The XPS results of N content were exhibited in Table 1. For NPC-800 900 and 1000, the N content is 1.96, 3.13 and 2.6 at%, respectively. The NPC-800 shows a low N content because of extremely high O proportion (up to 20.68 at%). As demonstrated in the literature, the N and O doping can effectively improve the pseudocapacitance and wettability of carbon electrode materials in the aqueous electrolyte.^22,42,43^

**Table 1:** Physical and electrochemical properties of NPC materials

Sample	*S*_BET_ (m^2^ g^−1^)	*V*_pore_ (cm^3^ g^−1^)	Ultimate analysis			XPS			*C*_spe_ (F g^−1^)
			C_wt%_	N_wt%_	H_wt%_	C_at%_	N_at%_	O_at%_	
NPC-800	589	0.32	80.95	1.04	0.915	77.36	1.96	20.68	195
NPC-900	1234	0.59	82.72	1.13	0.654	80.78	3.13	16.09	213
NPC-1000	1209	0.57	86.41	0.96	0.303	87.01	2.6	10.4	175

**Fig. 4:**
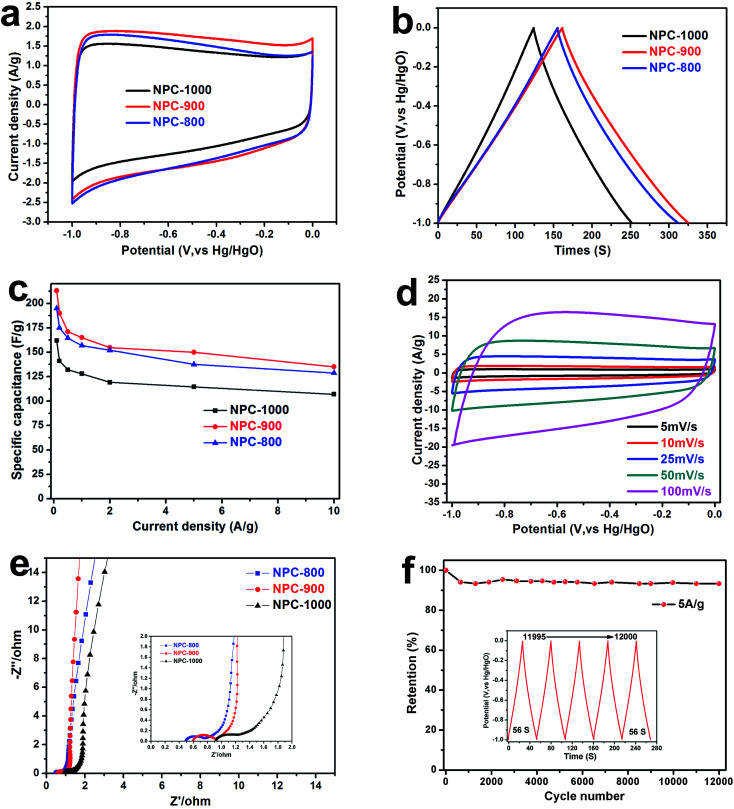
(a) CV curves of NPC-800, NPC-900 and NPC-1000 at scan rate of 10 mV s^−1^. (b) GCD curves of NPC-800, NPC-900 and NPC-1000 at the current density of 1 A g^−1^. (c) The specific capacity of NPC-800, NPC-900 and NPC-1000 at various current densities. (d) CV curves of NPC-900 at different scan rates. (e) Nyquist plots of NPC-800, NPC-900 and NPC-1000. (f) Cycle performance for NPC-900 at 5 A g^−1^.

The *N*,*O*-functionalities and hierarchical nanoporous structure of NPC are beneficial for supercapacitors applications as well. Fig. 4 shows the electrochemical performance of NPC carbons calcined at different temperatures in a three-electrode supercapacitor setup tested in 6 M KOH electrolyte. The CV curve of NPC-800, NPC-900 and NPC-1000 at a scan rate of 10 mV s^−1^ was showed in Fig. 4a. The NPC-900 obtained the biggest CV area in all samples, indicating that NPC-900 could make a better performance than the others in supercapacitors. Fig. 4b shows the GCD curves of NPC materials with different carbonization temperature electrodes at a current density of 1 A g^−1^. The NPC-900 shows the longest charge–discharge time than others, demonstrating the higher capacitance and has been fully tallies with the above CV results. The gravimetric capacitances of the NPC-800, NPC-900 and NPC-1000 samples were calculated at various current densities ranging from 0.1 to 10 A g^−1^ (Fig. 4c), resulting in a value as high as 213 F g^−1^ of NPC-900, at a current density of 0.1 A g^−1^. Moreover, the high specific capacitance of the NPC-900 can be still obtained at a much higher current density (135 F g^−1^ at 10 A g^−1^). The CV curves of NPC-900 at a scan rate ranging from 5 to 100 mV s^−1^ are exhibited in Fig. 4d. The rectangular shape maintained at a high scan rate of 100 mV s^−1^ reveals the quick charge transfer of the EDLC, fully exhibiting capacitive performance of NPC-900. The IR drops curve of NPC-900 is showed in Fig. S4.† The small IR drops (0.0778 V) even at a high current density of 10 A g^−1^ demonstrates that the NPC-900 electrode has excellent reversibility and small internal resistance. Fig. 4e shows the Nyquist plots of NPC-800, 900 and 1000 electrode-based supercapacitors in the frequency ranging from 10 kHz to 10 mHz. The Nyquist plot expanded in the high frequency region is presented in the inset. The semicircle diameter of NPC-800, NPC-900 and NPC-1000 was 0.3, 0.32 and 0.4 Ω, respectively, illustrating the good conductivity of the materials. Moreover, and the short Warburg-type lines (in the low frequency) indicate the low resistance of ion transportation and high ion diffusion efficiency for NPC, especially for NPC-900. As shown in Fig. 4f, NPC-900 shows an excellent cycle life with less than 6.6% capacitance loss after 12 000 cycles (140 F g^−1^ at 5 A g^−1^). Fig. S5† shows the electrochemical performance of NPC-900 when the loading mass is 8 mg cm^−2.^ The CV curves (Fig. S5a†) and GCD curves (Fig. S5b†) show an excellently reversible and high capacitance in discharge–charge process, respectively. Fig. S5c† shows a specific capacitance with different loading mass in NPC-900 electrode. Even at high loading mass is 8 mg cm^−2^ in an electrode, the NPC-900 still obtaining high capacitance (196 F g^−1^). Moreover, a small resistance (Fig. S5d†) at high loading mass are also proved the excellent electrochemical performance of NPC-900.

In conclusion, the best performance was obtained when NPC-900 acts as electrode materials in supercapacitors. Compared to other NPC samples, the NPC-900 shows the biggest EDLC because of the highest SSA of NPC-900 (1234 m^2^ g^−1^) in all samples. However, NPC-1000 shows a low specific capacitance (162 F g^−1^ at 0.1 A g^−1^) at the high SSA (1209 m^2^ g^−1^). Thus, the SSA is not the only impact factor of capacitance. In comparison to NPC-800 capacitance, it could be found that *N*,*O*-functionalities of NPC is another impact factor of capacitance. NPC-800 shows up to 195 F g^−1^ specific capacitance at 0.1 A g^−1^ current density because of *N*,*O*-enriched. In the aqueous electrolyte, the introduction of *N*,*O* heteroatom not only improves the surface wettability of NPC electrode material, but also enlarges the whole capacitance due to the existence of pseudocapacitance (N-doped).^10,15^ With the calcination temperature increasing, the *N*,*O*-functional group content decreased. When calcination temperature increasing up to 900 °C, a high SSA and reasonable amount of *N*,*O*-doping were created, which contributed to the high specific capacitance. Due to the extremely high calcination temperature (1000 °C) of NPC materials, the content of *N*,*O*-functional group drastically decreases (N: from 3.13 to 2.6; O: from 16.9 to 10.4) as well as pore structure collapses resulting in low capacitance. However, due to the over low SSA (only 589 m^2^ g^−1^) for NPC-800 with good conductivity, NPC-800 hardly obtain the better performance than NPC-900 (1234 m^2^ g^−1^). All of the above studies show that the high capacitance of NPC-900 exhibits the best results that could be due to the synergy work of high SSA, functionalized heteroatoms and conductivity.

**Fig. 5:**
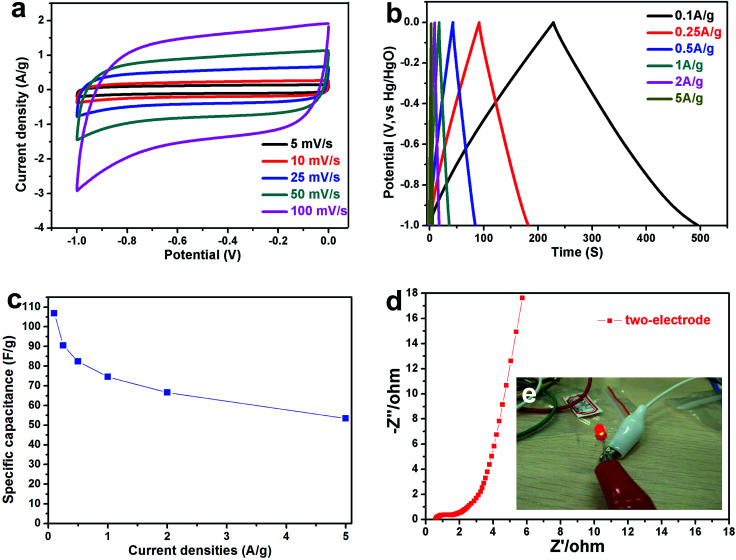
CV curves (a) and GCD curves (b) for NPC-900 tested in a two electrode system. (c) Rate performance of NPC-900 at different current densities in two-electrode system. (d) Nyquist plots of NPC-900 in a two-electrode system. (e) The digital image of LED with light up by a device of two supercapacitors connected in series.

Next, the best performance sample (NPC-900) has also been investigated in symmetric supercapacitor with identical amount of active materials on both electrodes. The electrochemical performances of NPC-900 were evaluated as shown in Fig. 5.

Fig. 5a shows the CV curves of NPC-900 using a two-electrode cell. As shown in Fig. 5a, the device exhibits a rectangular CV shape at all scan rates with rapid current responses upon voltage reversal (from 5 mV s^−1^ to 100 mV s^−1^). The linear GCD curves at all current densities demonstrated the high-rate responses of the device.^27^ The obtained specific capacitances of the device are 107, 90, 82, 74, 67, and 53 F g^−1^ at the current densities of 0.1, 0.25, 0.5, 1, 2, and 5 A g^−1^, respectively (Fig. 5c). Moreover, the excellent conductivity was further proved by EIS as shown in Fig. 5d (the semicircle diameter was about 1.1 Ω). An energy storage device with two supercapacitors connected in series was fabricated. Such a simple energy storage device could power a commercial red light-emitting diode (LED) as shown in Fig. 5e.

Thus, it has proved the potential commercial value of NPC materials in energy storage. Compared to the previous reported carbon materials from biomass resource (Table 2), NPC obtained in this study shows good performance. Moreover, this facile approach may open a door for preparation of high surface area porous carbons for energy storage.

**Table 2:** Performance comparison of biomass carbon derived electrodes in supercapacitors

Materials	Activation agent	*S*_BET_ (m^2^ g^−1^)	Test system	Electrolyte	Current density/scan voltage	*C*_spe_ (F g^−1^)	Ref.
Wood	CO_2_	—	3E	1 M Na_2_SO_4_	1 m A cm^−2^	118.7	44
Cotton	NaOH/urea	584.49	3E	3 M KOH	0.3 A g^−1^	221.7	34
Lignocelluloses	H_3_PO_4_	1135	3E	1 M H_2_SO_4_	0.05 A g^−1^	176	45
Fungi	Hydrothermal	80	3E	6 M KOH	5 mV s^−1^	196	46
Cassava peel waste	KOH + CO_2_	1352	3E	0.5 M H_2_SO_4_	10 A g^−1^	153	47
Water hyacinth	KOH	1010	3E	30 wt% KOH	—	179.6	48
Cow dung	KOH	2000	2E	Et_4_NBF_4_	1 A g^−1^	117	49
Firewood	Na_2_CO_3_ K_2_CO_3_	818	3E	1 M H_2_SO_4_	0.2 A g^−1^	189	50
Boiled coffee beans	CaCl_2_	550	2E	1 M MeEt_3_NBF_4_	0.4	93.4	51
Rootof multibract raspberry	Self-activation	1234	3E	6 M KOH	0.1 A g^−1^	213	Our work
			2E	6 M KOH	0.1 A g^−1^	107	

## 4. Conclusion

In summary, we report a facile synthesis of 3D sheet nanoporous carbon materials from plant, using trunk inner substance of ROMR as the precursor. These nanoporous carbon materials show high SSA (1234 m^2^ g^−1^) and large pore volume (0.59 cm^3^ g^−1^) without the need of complicated processes (*e.g.* activation, pre-treatment), except for calcination at 900 °C for 2 h. Moreover, these materials exhibited high specific capacitance (up to 213 F g^−1^ at 0.1 A g^−1^) and stable cycle performance (6.6% loss over 12 000 cycles) in three-electrode configuration. The high electrochemical performance was obtained due to the coherent work of high SSA and functionalized heteroatoms. Furthermore, the high capacitance (107 F g^−1^ at 0.1 A g^−1^) could also be obtained in a supercapacitor device, which further illustrated the fact that the nanoporous carbon materials, obtained with high yield, environmental friendliness, low-cost and being readily available, are appropriate for being used in energy storage devices.

## Conflicts of interest

There are no conflicts to declare.

## Supplementary Material

RA-008-C7RA12525A-s001
