# Supplementary material for: Facile synthesis of high-surface-area nanoporous carbon from biomass resources and its application in supercapacitors
Source: RSC Adv. 2018 Jan 9;8(4):1857–65. doi: 10.1039/c7ra12525a (PMC9077209; doi:10.1039/c7ra12525a)
Supplement: RA-008-C7RA12525A-s001 [file RA-008-C7RA12525A-s001.pdf]

Electronic Supplementary Material (ESI) for RSC Advances.
This journal is © The Royal Society of Chemistry 2017

Facile synthesis of high-surface-area nanoporous carbon from biomass resource and their application in supercapacitors

Yuechao Yao[a], Qi Zhang[c], Peng Liu[a], Liang Yu[a], Lin Huang[a,b], Shaozhong Zeng[a,b], Lijia Liu[a,b], Xierong Zeng[a], Jizhao Zou[*,a]

Fig. S1 The introduction of Root Of Multibract Raspberry.

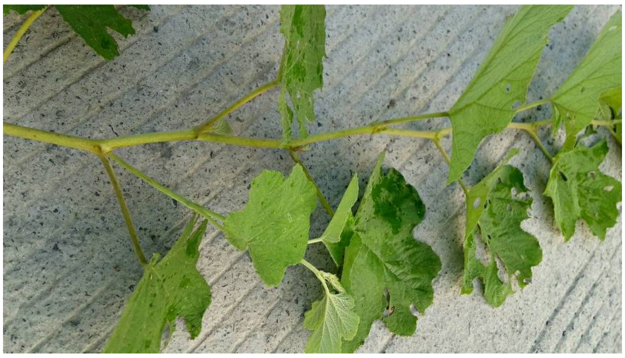

Title: Root Of Multibract Raspberry
Latin plant animal Mineral name: Rubus Multibracteatus Lévl.Et Vant.
The original form of Root Of Multibract Raspberry shrubs, 2-3m high. Flowering from April to June, fruit period from August to September.
Habitat distribution of the ecological environment: born at an altitude of 700-2500 m hillside and valley shade shrub or forest edge and roadside.
Distribution of resources: distributed in China's Guangdong, Guangxi, Yunnan, Guizhou province.
Link information: http://www.xjishu.com/yiyao/zy/28344.html.

Table S1 the various element content of Root Of Multibract Raspberry.

Element	Ray-type	Apparent concentration	k ratio	wt%	wt% Sigma	Standard sample label
C	K	20.55	0.20549	58.23	0.18	C Vit
O	K	14.77	0.04969	39.86	0.18	SiO2
Al	K	0.08	0.00061	0.16	0.02	Al2O3
Cl	K	0.21	0.00181	0.39	0.02	NaCl

K	K	0.48	0.00410	0.87	0.03	KBr
Ca	K	0.26	0.00235	0.49	0.03	Wollastonite
Total:				100.00		

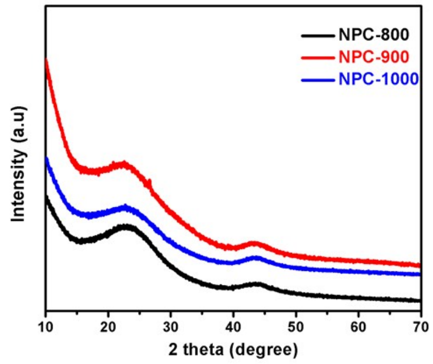

Fig. S2. The XRD patterns of NPC for different calcination temperatures.

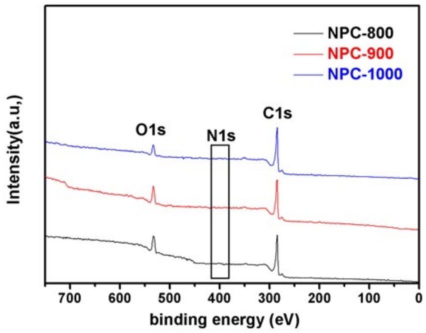

Fig. S3. The XPS spectrum of NPC-800,900 and 1000.

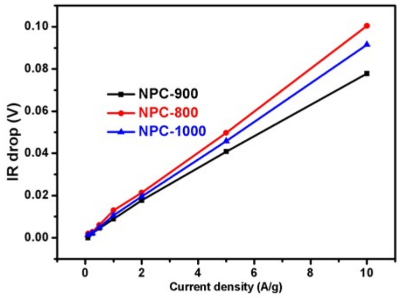

Fig. S4. The IR drops curves of NPC800, 900 and 1000.

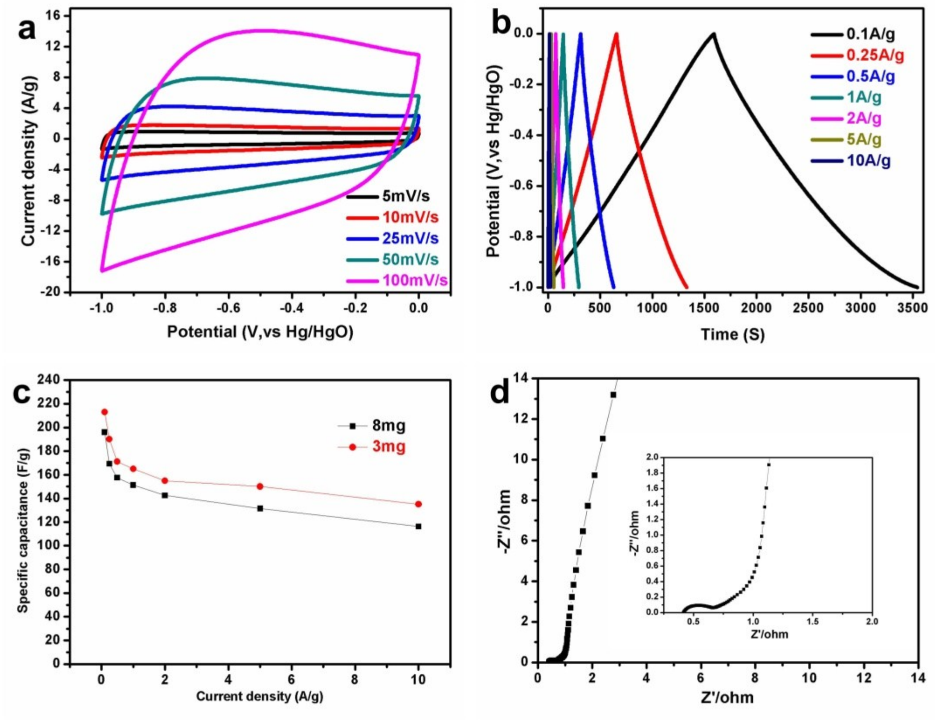

Fig. S5 (a) CV curves of NPC-900 (8 mg cm^{-2}) at different scan rates. (b) GCD curves of NPC-900 (8 mg cm^{-2}) at the different current densities. (c) The specific capacity of NPC-900(8,3 mg cm^{-2}) and at various current densities. (d) Nyquist plots of NPC-900 (8 mg cm^{-2}).
